# Chitosan–Selenium Nanoparticle (Cs–Se NP) Foliar Spray Alleviates Salt Stress in Bitter Melon

**DOI:** 10.3390/nano11030684

**Published:** 2021-03-09

**Authors:** Morteza Sheikhalipour, Behrooz Esmaielpour, Mahdi Behnamian, Gholamreza Gohari, Mousa Torabi Giglou, Pavla Vachova, Anshu Rastogi, Marian Brestic, Milan Skalicky

**Affiliations:** 1 Department of Horticultural Sciences, Faculty of Agriculture and Natural Resources, University of Mohaghegh Ardabili, Ardabil 56199-11367, Iran; p.sheikhalipour@uma.ac.ir (M.S.); mbehnamian@uma.ac.ir (M.B.); mtorabi@uma.ac.ir (M.T.G.); 2 Department of Horticultural Sciences, Faculty of Agriculture, University of Maragheh, Maragheh 55181-83111, Iran; gohari.gh@maragheh.ac.ir; 3 Department of Botany and Plant Physiology, Faculty of Agrobiology, Food and Natural Resources, Czech University of Life Sciences Prague, Kamycka 129, 16500 Prague, Czech Republic; vachovap@af.czu.cz (P.V.); marian.brestic@uniag.sk (M.B.); 4 Department of Ecology and Environmental Protection, Poznan University of Life Sciences, Piątkowska 94, 60-649 Poznan, Poland; anshusls@gmail.com; 5 Department of Plant Physiology, Slovak University of Agriculture, Nitra, Tr. A. Hlinku 2, 94901 Nitra, Slovakia

**Keywords:** abiotic stress, antioxidant enzymes, bitter melon, fruit quality, nanocomposites, nanotechnology

## Abstract

Salt stress severely reduces growth and yield of plants. Considering the positive effects of selenium (Se) and chitosan (Cs) separately against abiotic stress, in these experiments, we synthesized chitosan–selenium nanoparticles (Cs–Se NPs) and investigated their ability to reduce the negative effects of salt stress on growth and some biochemical parameters of bitter melon (*Momordica charantia*). Bitter melon plants were grown at three NaCl salinity levels (0, 50, and 100 mM) and a foliar spray of Cs–Se NPs (0, 10, and 20 mg L^−1^) was applied. Some key morphological, biochemical, and physiological parameters in leaf samples and essential oil from fruit were measured at harvest. Salinity decreased growth and yield while foliar application of Cs–Se NPs increased these critical parameters. Furthermore, Cs–Se NPs enhanced bitter melon tolerance to salinity by increasing antioxidant enzyme activity, proline concentration, relative water content, and K^+^, and decreasing MDA and H_2_O_2_ oxidants and Na aggregation in plant tissues. Yield was also improved, as the highest amount of essential oils was produced by plants treated with Cs–Se NPs. Generally, the greatest improvement in measured parameters under saline conditions was obtained by treating plants with 20 mg L^−1^ Cs–Se NPs, which significantly increased salinity tolerance in bitter melon plants.

## 1. Introduction

Salt stress can be a severe problem for plants because it decreases growth and production. Salinity alters a plant’s metabolism and physiology by reducing uptake of water and nutrients, and inhibiting growth and photosynthesis. Abdelrahman et al. [1] reported that salt stress affected more than 6% of global arable land. Saline conditions cause stress from changes in osmolality, ion toxicity, and overproduction of harmful reactive oxygen species (ROS) [2]. ROS are reactive molecules that cause oxidative stress, lipid peroxidation, damage to proteins and nucleic acids, inactivation of antioxidant enzymes [3], and ultimately cell death [4].

Selenium (Se) is a beneficial nutrient for plants that increases growth and yield, and has antioxidant activity [5]. Selenium is also used in fertilizers and as a fungicide [6]. In 1957, Schwarz and Foltz proved that selenium was an essential element for living organisms [7]. Selenium was found to increase plant growth at low concentrations but decrease it at high [8]. Selenium is a necessary element for animals including humans and has several beneficial roles in plants. Se application improved salinity tolerance in sorrel (*Rumex acetosa* L.) seedlings by maintaining mitochondrial integrity, chloroplast ultrastructures, and increasing photosynthesis [9]. Diao et al. [10] stated that Se application prevented damage to photosynthesis in tomato plants under salt stress. Se has a positive effect on photosynthesis and antioxidant defense systems. Hawrylak-Nowak [11] observed that exogenous Se application to cucumbers under salt stress led to enhanced growth, increased proline content and photosynthetic pigments, and improved salt tolerance. Se can also increase the concentration of secondary metabolites in plants. For example, Handa et al. [12] showed that application of Se to *Brassica juncea* L. increased expression of the *PAL* gene and production of secondary metabolites.

Nanotechnology is a promising new area of bioengineering utilizing the unique characteristics of nanoscale particles with diameters less than 100 nm. Nanoparticles (NPs) are excellent carriers for delivery of various drugs and compounds and they have found application in a novel treatment for increasing salinity tolerance in plants [13]. Gohari et al. [14] showed that NP application, in addition to increasing growth and antioxidant status, enhanced production of secondary metabolites in medically important plants under salt stress. Hussein et al. [15] showed that application of Se NPs improved growth and antioxidant defenses in groundnut cultivars under stress condition. As well as Morales-Espinoza et al. [16] reported that application of Se NP increased growth and antioxidant compound (enzymatic and non-enzymatic) in tomato under salt stress. Djanaguiraman et al. [17] stated that Se NPs had high solubility, low cytotoxicity, and excellent bioactivity in comparison with metal-based Se compounds (selenate and selenite). In this regard, application of Se NPs improved root regeneration and organogenesis in tobacco (*Nicotiana tabacum*), whereas selenate inhibited both processes, under controlled growth conditions [18]. Metal-based nanomaterials are non-degradable and highly reactive and may have unanticipated health risks due to their persistence in the food chain. Numerous studies have demonstrated the safety and efficacy of the crustacean shell derivative, chitosan (Cs), as a matrix for encapsulating and sequestering bioactive compounds [19]. Cs-NPs have been used as carriers for slow release and adsorption fertilizers, pesticides, herbicides, and plant growth regulators. The use of Cs-NPs for encapsulating and delivering bioactive compounds can protect plant cells from the dangerous effects of burst release. Cs-NPs can also shield biomolecules from damage by temperature, light, and pH. Encapsulation within a Cs matrix as micro- or nanoscale carriers has great potential in agriculture [20]. The use of Cs for coating Se NPs can prevent potentially harmful effects on plants and the consumers of plant products. To this end, our research group has successfully developed biodegradable chitosan-based nanomaterials functionalized with selenium.

Bitter melon (*Momordica charantia* L.) belongs to the Cucurbitaceae family. It is cultivated as a medicinal plant as well as a vegetable and contains some sixty phytochemicals with activity against human diseases such as cancer, diabetes, and AIDS [21]. During its cultivation in various regions, bitter melon may be exposed to various stresses, one of the commonest of which is excess salt from soil or irrigation with saline water [22,23,24]. The effects of chitosan-selenium nanoparticles (Cs–Se NPs) on salinity in plants has not been studied. Therefore, our group synthesized chitosan-selenium nanoparticles (Cs–Se NPs) for the first time and this study was performed to investigate the effects of a foliar spray of chitosan-selenium nanoparticles (Cs–Se NPs) on growth, biochemical, physiological properties, and essential oil content of *M. charantia* grown under saline conditions.

## 2. Materials and Methods

### 2.1. Plant Material and Growing Conditions

The experiment was conducted in the research greenhouses of the Faculty of Agriculture, of Mohaghegh Ardabili University (46°16′ E, 37°23′ N, altitude 1485 m), as a factorial experiment using a random design.

Seeds of bitter melon (*Momordica charantia* L. cv. Palee F_1_) were provided by the Victoria Companies in India. For sterilization, seeds were placed in sodium hypochlorite solution (1%) for 5 min then pre-germinated in the dark at 25 °C for 48 h. After one week, the germinated seeds were planted in cultivation trays containing coco peat. The trays were kept in a growth chamber at 28/22 °C (day/night) and 62–80% relative humidity under natural light. Seedlings were transferred to main pots (40 × 15 cm) containing coco peat and Perlite (2:1, *v*/*v*) after appearance of two true leaves and irrigated each day uniformly for one week then fertigated with half-strength Hoagland’s nutrient solution daily. Thereafter, the plants were continuously watered with full-strength Hoagland’s supplemented with NaCl at concentrations of 0, 50, and 100 mM. Plants were sprayed with chitosan-selenium nanoparticles (Cs–Se NPs) at concentrations of 0, 10, and 20 mg L^−1^ applied once a week during the growth period (six times). All treatments were dispersed in Deionized water (DIW).

### 2.2. Preparation of Chitosan-Selenium Nanoparticles (Cs-Se NPs)

Chitosan (Cs) with 75–85% deacetylation and 310–375 kDa molecular weight, sodium selenite (Se), and tripolyphosphate (TPP) were obtained from Sigma-Aldrich Co (St Louis, MO, USA). Other chemicals used in this project were all analytical grade, and used without further purification. Deionized water (DIW) was used for this investigation. Cs–NPs were prepared according to a published method [25]. Briefly, a Cs solution was obtained by adding 0.5 g of Cs powder to 25 mL of 1% (by wgt) acetic acid with continuous stirring for 3 h at room temperature. Separately, 0.1 g of sodium selenite was added to 15 mL of DIW and dissolved by shaking vigorously. The Se solution was then added to the Cs solution. The ratio of Cs to TPP by weight was 2.5:1, so 0.2 g of TPP was dissolved in 10 mL of DIW and slowly added to the Cs-Se solution. The coagulum of TPP–crosslinked Cs was left to stir overnight at room temperature and subsequently, washed with excess DIW to remove unreacted starting materials. Lastly, the Cs–Se NPs were lyophilized.

### 2.3. Plant Growth, Fruit Parameters, and Relative Water Content (RWC) in Leaves

The fresh weight (FW) of shoots and leaves was recorded at harvest, and shoot and root dry weight (DW) was measured after samples were oven dried (UFP800, Memmert, Büchenbach, Germany) at 70 °C for 72 h. Unripe fruits were collected over eighty-five days, counted, and the yield and average weight determined. RWC of leaves in treated and untreated salt-stressed plants was determined by the method of Sairam and Srivastava [26]. Initially, fresh leaves were weighed for fresh weight (FW), then, turgid weight (TW) was measured after keeping leaf samples in DIW for 24 h. Lastly, the dry weight (DW) was determined after 24 h drying at 70 °C. RWC was determined by the formula:

% RWC = (FW − DW)/(TW − DW) × 100(1)

### 2.4. Photosynthetic Pigments and Gas Exchange Capacity

Chlorophyll a and b, total chlorophyll, and carotenoids were isolated from fresh leaves using 80% (*v*/*v*) acetone. After centrifugation (15,000× *g* for 5 min at 25 °C), the absorbance of the resulting extracts was determined spectrophotometrically at 470, 646 and 663 nm (UV-1800 Shimadzu, Kyoto, Japan) and the concentration of photosynthetic pigments was determined by the following equations from Arnon [27]:

Chl a = (12.47 × A663) − (3.62 × A 645)(2)

Chl b = (25.06 × A645) − (6.5 × A663)(3)

Carotenoids = (1000 × A470) − (1.29 Chl a − 53.78 Chl b)(4)

Measurements of net photosynthetic rate (P_N_) were carried out on the of adult leaves, using an Infrared (IR) gas analyzer (LI-6400T, Li-Cor Inc., Lincoln, NE, USA), with red/blue light source (6400-02B) [28].

### 2.5. Leaf Sodium and Potassium Content

The concentration of sodium and potassium was measured in leaves after digestion of 0.10 g of oven-dried tissues in HNO_3_ at 110 °C. The Na and K in the digests was quantified by atomic absorption spectrometry (AA-7000 from Shimadzu, Kyoto, Japan)

### 2.6. Leaf Proline and Total Soluble Carbohydrates

Leaf proline and total soluble carbohydrates were measured using published methods [29,30], respectively.

### 2.7. Malonaldehyde (MDA) and Hydrogen Peroxide (H_2_O_2_) Content and Electrolyte Leakage (EL)

Lipid peroxidation was measured using the amount of malondialdehyde (MDA) by the Heath and Packer [31] method. The samples of fresh leaf tissue (0.3 g) were ground in 20% trichloroacetic acid and centrifuged at 13,000 rpm for 15 min and 4 cm^3^ of 20% TCA were added into 1 cm^3^ of gained supernatant. The mixtures were heated for 30 min in the hot water bath (95 °C). Thereafter, immediately cooled in an ice bath. Malondialdehyde content was determined at two wavelength of 532 and 600 nm. To calculate the MDA concentration a molar absorption coefficient of 155 mM^−1^ cm^−1^ was used. MDA content was expressed as nmol g^−1^ FW.

The hydrogen peroxide (H_2_O_2_) levels was determined accordance with Allen [32] method. Leaf sample (0.2 g) was crushed in an ice bath with 3 mL 0.1% TCA and centrifuged at 20,000 rpm for 15 min and afterwards 500 µL resulting supernatant was mixed with 500 µL 10 mM potassium phosphate buffer (PH 7.0) containing 2 M potassium iodide. The reaction mixture was placed in dark at room temperature for one hour. H_2_O_2_ solutions were prepared at concentrations ranging from 2 to 10 mM and standard graph was plotted. The amount of H_2_O_2_ was measured spectrophotometry at 390 nm. H_2_O_2_ content was expressed as nmol g^−1^ FW.

Electrolyte leakage through cell membranes is a characteristic of salt stress in plants. Fresh bitter melon leaves were finely chopped and excess electrolytes were removed by washing three times with DIW. Samples were suspended in 10 mL of DIW at room temperature (25 °C) and shaken 24 h at 120 rpm. The primary electrical conductivity (EC1) was first measured by EC meter, and then the samples were autoclaved at 100 °C for 2 h to release all electrolytes from the tissue. Samples were cooled to room temperature (RT) and electrical conductivity (EC2) was measured. Percent electrolyte leakage was calculated as:

% EL = EC1/EC2 × 100(5)

### 2.8. Antioxidant Enzymes

Samples of leaves were weighed 0.5 g, homogenized in 5 mL of 0.05 M phosphate buffer (1 mM EDTA, 1% PVP, pH 7.8), and the homogenates were centrifuged at 12,000× *g* for 20 min at 4 °C. The supernatants were withdrawn and used for the determination of peroxidase (POD), ascorbate peroxidase (APX), catalase (Cat), and superoxide dismutase (SOD) activity. The activity of POD was measured by the method of Rao [33], Cat by Aebi [34], SOD by Giannopolitis and Ries [35], and APX by the method of Nakano and Asada [36].

### 2.9. Fruit Quality and Biochemical Parameters

#### 2.9.1. Total Phenols, Flavonoids, Ascorbic Acid, Anthocyanin, and Radical Scavenging Activity (DPPH)

Phenols were extracted from 0.2 g frozen fruit material, which was homogenized with 2 mL methanol 80% (*v*/*v*) in a cooled mortar at 4 °C. This was then centrifuged at 4 °C and relative centrifugal force (RCF) at 12.000 for 20 min. The supernatants obtained were used for the assays. Total phenol concentration assayed using the Folin–Ciocalteu reagent, as described by Chun et al. [37]. Total phenolic content was expressed as mg gallic acid 10g^−1^ of FW. The total flavonoid content of fruit methanolic extract was determined colorimetrically as described by Zhishen et al. [38] with some modifications. All values were expressed in mg of rutin g^−1^ of fruit fresh weight (FW). Ascorbic acid was determined on the basis of coupling 2,4-dinitrophenylhydrazine with the ketonic groups of dehydroascorbic acid through the oxidation of ascorbic acid by 2,6-dichlorophenol-indophenol sodium salt dihydrate (103028 Merck, Hamburg, Germany) to form a yellow orange color in acidic conditions as described by Suntornsuk et al. [39]. Ascorbic acid content was expressed as mg 10g^−1^ of fruit fresh weight (FW). The anthocyanin content was measured spectrophotometrically as described previously Sakamoto and Suzuki [40], with modifications. Fresh fruit were promptly dried in an oven at 90 °C for 1 day. Dried fruit were weighed (about 15 mg) and soaked in 1 mL of methanol containing 1% HCl and were incubated at 95 °C for 15 min. The sample was then cooled to room temperature. After the removal of the fruit, absorbance of the supernatant was measured at 533 nm, and a standard calibration curve was prepared using cyanidin-3-glucoside. Anthocyanin content was expressed as µM g^−1^ of fruit fresh weight (FW). Total antioxidant activity was determined following the method described by Suja et al. [41], preparing a 0.1 mM solution of 2.2-diphenyl-1-picrylhydrazyl (DPPH) in absolute ethanol. The antioxidant activity (%) was determined using the following formula:

A0−A1/A0 × 100(6)

A0 = the absorbance of control; A1 = the absorbance of standard

#### 2.9.2. Essential Oils and Associated Compounds

The contents of essential oils (mL 100 g^−1^ of FW) were measured in a Clevenger-type apparatus. Fresh fruits of *Momordica charantia* L. were hydro-distilled four h at a distillation rate of 3.0–3.5 mL min^−1^, in five replicates. The oils were dried over sodium sulfate (anh) and held at 4–6 °C. Volatile compounds were analyzed by gas chromatography on a Varian 450 GC, 240 MS (VF-5 MS column) with injector and oven temperature at 250 °C and 200 °C, respectively. The heating ramp was programmed for 10 °C/min and injection was performed in a split ratio of 200 and a volume of 10 µL. The carrier gas flow was 1.0 mL min^−1^ [42]. The quantification of components was done by relative peak areas calculation. Relative peak areas were calculated by dividing the peak area for compound by the total peak areas for the entire compounds detected and expressing this value as percent. Oleic acid, stigmasterol, cis-9-hexadecenal, cucurbitacin, pentadecyne, and gentisic acid were identified by gas chromatography. Moreover, a gradient HPLC system with ODS C18 column (250 mm × 4.6 mm, 5 µm) was used. The samples were mixed with 80% (v/v) ethanol and incubated at 25 °C for 60 min to complete the extraction of compounds. The mobile phase components, methanol: water (98:2) were filtered through a 0.2 µm membrane filter before use and the flow rate was 1 mL/min. Column temperature was 27 °C. The 20-µL samples were injected with a Hamilton syringe and isocratic elution was performed [43]. Charantin, momordin, momordicoside, momordicin, karaviloside, and cucuritan were calculated based on the peak area of the standard compounds and the calibration curve.

### 2.10. Statistical Analysis

This study was arranged in factorial format with a completely randomized block design of nine treatments and three replicates. Statistical analysis of results was performed with statistical software ver. 12 (StatSoft Inc., Tulsa, OK, USA). Data were subjected to factorial ANOVA and means were compared with Tukey’s multiple range test (TMRT) (significance at *p* < 0.05). Canoco 5 software [44] was used for principal component analysis (PCA). Data normalization can be used for fluorescence [45], but we used logarithmic transformation. Data were calculated from centered, but not standardized, data. PCA was suitable for evaluating differences between Cs–Se NP treatments and tolerance of stress.

## 3. Results and Discussion

### 3.1. Synthesis and Characterization of Cs–Se NPs

**Figure 1:**
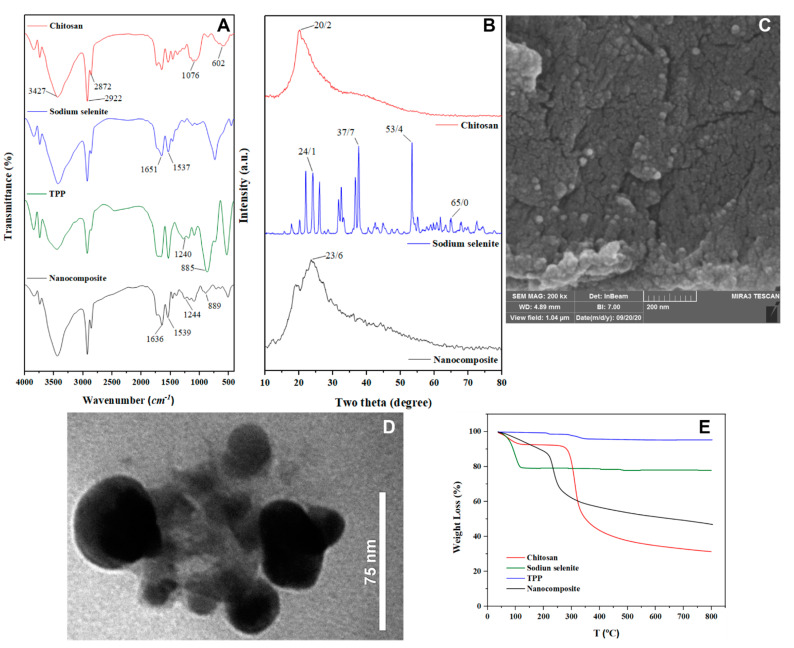
FT-IR (**A**), XRD (**B**) SEM (**C**), TEM (**D**) and TGA (**E**) analysis of starting materials and chitosan-selenium nanoparticles (Cs–Se NPs).

FT-IR spectrometry was used to study the composition and structural properties of the components and the nanocomposites (Figure 1A). The results confirmed the structural features of chitosan: –OH and –NH_2_ stretching at 3426 cm^−1^, CH stretching at 2922 cm^−1^ and 2872 cm^−1^, –NH_2_ stretching at 1601 cm^−1^, C–O–C stretching at 1076 cm^−1^ and pyranoside ring stretching vibration at 601 cm^−1^. The spectrum revealed a sharp peak at 888 cm^−1^ from Se-O stretching in the sodium selenite fingerprint region, matching published reports [46]. The FT-IR data can be used for qualitative identification of Se in NPs. The –NH_2_ bending vibration shifts from 1601 cm^−1^ to 1542 cm^−1^ and a new peak at 1637 cm^−1^ indicates interaction between NH_3_^+^ groups of Cs and TPP in the NPs. This shows that sodium selenite was efficiently incorporated into the Cs NPs. The X-ray diffraction pattern of Cs-Se NPs is shown in Figure 1B. There are three prominent peaks of sodium selenite at 2θ = 24.1°, 37.7°, 53.4°, and 64.9°, which confirms a high level of crystalline structure. Cs shows one slightly broad peak at 2θ = 20.2°. After TPP crosslinking, the initial peaks remained although with lower intensity. New peaks at 18° and 24° appeared, indicating that the crystal lattice was being affected by ionic interactions. XRD peaks depend on crystal size, but the distinctive peaks of sodium selenite may overlap, perhaps because of well-dispersion of selenium into the nanocomposite matrix [47].

The morphological characteristics of Cs–Se NPs were imaged using TEM and SEM (Figure 1C,D). The results showed that the nanoparticles were spherical with an average size of 50 nm. The thermal behavior of Cs and Cs–Se NPs was investigated and Cs showed a characteristic TGA pattern (Figure 1E) with the initial stage corresponding to water loss at 95 °C (7.5% w/w loss) after which the Cs decomposed showing a 2-stage weight change. The carbonization inflection point was seen at the same temperature ending at 800 °C. The endothermic point at 210 °C results from water evaporation during the initial mass loss on the TGA curve. This peak and the contrasting thermograms of Cs and cross-linked Cs, are evidence that the exothermic event at 250 °C, coinciding with loss of cross-linked Cs, was linked to the bond breakage and carbonization of Cs cross-linked by TPP.

### 3.2. Influence of Cs–Se NPs on Plant Growth, Fruiting, and RWC

Salt stress inhibits bitter melon growth in terms of shoot and root FW and DW, stem height, and RWC (Table 1). High salinity significantly decreased shoot and root FW and DW, stem height and RWC by 62.81%, 41.28%, 55.67%, 55.57%, 69.26%, and 40.97%, respectively. Foliar application of Cs–Se NPs reversed the negative effects of salt stress on plant growth. There was also a dose response as 20 mg/L Cs–Se NPs had a larger positive effect on plant growth under saline conditions than 10 mg/L (Table 1). The results showed that application of Cs–Se NPs at 20 mg L^−1^ significantly improved shoot FW by 16.97% and 24.20%, shoot DW by 7.14% and 13.35%, root FW by 13.59% and 10.29%, root DW by 13.59% and 10.41%, stem height by 7.03% and 13.47%, and RWC by 13.46% and 27.53% under 50 and 100 mM NaCl, respectively (Table 1).

Results showed that salinity significantly reduced the number and weight of fruits and the overall yield but applying a foliar spray of Cs–Se NPs to salt-stressed plants significantly increased fruit count and total weight. The highest dose of Cs–Se NPs applied (20 mg L^−1^) gave the best results. High salinity (100 mM NaCl) significantly decreased yield (78.94%), fruit weight (50.70%), and number of fruits (57.14%) compared to non-saline conditions. Under conditions of treatment with 0, 50, and 100 mM NaCl, Cs–Se NPs (20 mg L^−1^) significantly increased yield by 11.80%, 25.00%, and 25.11%, fruit weight by 5.63%, 17.30%, and 22.85%, and number of fruits by 7.90%, 13.95%, and 10.00% (Table 1). Salinity causes osmotic stress and preservation of water balance is necessary for tolerance of osmotic stress. Cs–Se NP application improved RWC of plants under salt stress (Table 1). Thus, Se is able to maintain water homeostasis in plants under osmotic stress conditions.

In agreement with our results, Jiang et al. [48] showed that exogenous Se application improved growth of maize plants in high salt stress conditions. The effects of Se on increasing growth and yield of plants in saline soil depended on the plant species, the application method, the salinity level and the concentration of applied Se. Mozafariyan et al. [49] observed that application of 10 mg L^−1^ Se to tomato plants had a better effect on plants under saline conditions, while Jiang et al. [48] found that 20 mg L^−1^ Se worked best for maize. Applications of Se at relatively low doses can increase growth and yield of plants, but higher concentrations may be toxic [48]. In our experiments, Cs–Se NPs at 10 and 20 mg L^−1^ were used. Djanaguiraman et al. [17] reported that Se NPs were less toxic and more effective than bulk Se particles because of their higher mobility, solubility, and large surface area. In addition, Hernández-Hernández et al. [50] reported that chitosan itself was able to decrease salinity stress in tomato plants. Our results showed that the highest concentration of Cs–Se NPs (20 mg L^−1^) was most effective in increasing growth parameters of bitter melon plants under salt stress. In agreement with our finding, Morales-Espinoza et al. [16] in tomato, and Zahedi et al. [51] in strawberry reported that application of Se NPs (20 mg L^−1^) significant increased growth parameters under salinity condition.

In this study, severe salinity significantly reduced fruit yield and size of bitter melon. Selenium is a beneficial nutrient for plant [11]. Thus, selenium has positive effects on crops. Application of Na_2_SeO_3_ (20 µM) increased yield of eggplants (*Solanum melongena*) under salt stress [52]. Moreover, NPs have useful effect on different physiological parameters, such as root and shoot growth in plant. For example, application of Se NPs (265–530 µM) promoted root growth in tobacco tissue culture (*Nicotiana tabacum*) [18]. This may be related to the increase in phytochemical yield and fruit weight that we observed in Cs–Se NP-treated plants compared to non-treated controls. In agreement with our results, a previous study showed that application of Se NPs significantly increased the number of fruits, fruit weight, and yield in plants under salinity condition [16,51].

The results of this study showed that Cs–Se NPs were able to maintain water homeostasis in bitter melon plants under osmotic stress conditions. Se application to garlic seedlings under salt stress significantly improved the RWC [53]. Furthermore, Zahedi et al. [51] reported that application of Se NPs significant increased RWC in strawberry under salinity condition.

### 3.3. Effects of Cs-Se NPs on Photosynthetic Pigments and Gas Exchange Capacity under Salinity Stress Conditions

Salt stress led to a decline in the amount of Chl a and b, and total Chl and carotenoids. Severe salinity significantly decreased Chl a by 53.77%, Chl b by 56.94%, total Chl by 54.61%, and carotenoid content by 44.86%. Under non-saline and saline conditions, foliar spray with 20 mg L^−1^ Cs–Se NPs significantly increased Chl a by 9.88%, Chl by 8.35%, total Chl by 9.40%, and carotenoid content by 6.58% compared to control plants sprayed with NPs alone. Application of 20 mg L^−1^ Cs-Se NPs decreased the negative effects of salt stress and significantly improved Chl a by 20.25% and 31.38%, Chl b by 12.68% and 26.74%, total Chl by 6.82% and 30.11%, and carotenoid content by 15.54% and 16.82%, respectively, under 50 and 100 mM NaCl (Table 2). Severe salinity significantly decreased the net photosynthetic rate, Pn, by 24.37% relative to non-saline conditions. However, spraying with 20 mg L^−1^ Cs–Se NPs significantly increased Pn by 10.02%, 7.80%, and 11.13%, respectively, in non-saline, 50, and 100 mM NaCl conditions (Table 2).

Our results show that salinity reduced growth of bitter melon plants by decreasing the photosynthetic pigments, Chls and CARs. Saha et al. [54] reported that chlorophylls were very important in plants because of their involvement in photosynthetic potential and capacity [55]. In this study, foliar application of Cs-Se NPs reversed the decrease in chlorophyll and carotenoid levels caused by salt stress. Increased ethylene production under saline conditions could downregulate chlorophyll biosynthesis [56], activate chlorophyllase [57], and cause instability of the protein–pigment complexes [58]. Sultana et al. [57] also reported lower carotenoid levels because of degradation of β-carotene and reduced formation of zeaxanthin under salt stress. Se might accelerate Chl biosynthesis by facilitating electron transport in the respiratory chain and respiration [59]. Padmaja et al. [60] reported that Se can interact with porphobilinogen deaminase and 5-aminolevulinic acid dehydratase and thereby play a role in chlorophyll biosynthesis. Application of Cs–Se NPs to bitter melon plants improved photosynthetic capacity and growth by increasing Chls and CARs under salt stress conditions. Na_2_SeO_4_ application decreased chlorophyll degradation in garlic exposed to increased salinity [53]. Moreover, Zahedi et al. [51] and Morales-Espinoza et al. [16] reported that 20 mg L^−1^ Se NP foliar spray significantly increased Chl a, Chl b, and carotenoid on strawberry and tomato plants under non-saline and salinity stress condition. Similar to this reports, in this study Spray Cs–Se NPs increase the concentration of photosynthetic pigments in salt stress condition.

### 3.4. Effects of Cs-Se NPs on Leaf K and Na Concentration under Saline Conditions

**Figure 2:**
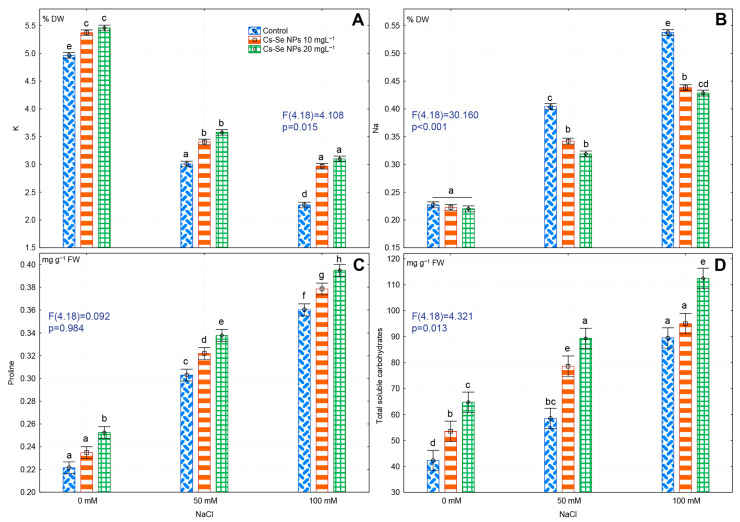
Effect of increasing concentration of Cs–Se NPs on K^+^ (**A**), Na^+^ (**B**), proline (**C**) and total soluble carbohydrate (**D**) content of bitter melon (*Momordica charantia*) leaves under salinity stress. The SDs denoted by lower-case letters in the same treatment indicates significant differences at *p* < 0.05 according to Tukey’s post hoc test.

Increased salinity leads to significantly increased Na and decreased K content in the leaves of bitter melon. Severe salinity significantly decreased K by 54.43% and increased Na by 57.72% compared to non-saline controls. Under salt stress, Cs–Se NP application significantly increased K content and decreased Na content compared to untreated plants. Cs–Se NPs also significantly increased K content in plants grown under non-saline conditions. Application of 20 mg L^−1^ Cs–Se NPs significantly increased K level by 8.99%, 15.96%, and 26.86% respectively, with no salt and with exposure to 50 and 100 mM NaCl (Figure 2A). Exogenous 20 mg L^−1^ Cs–Se NPs decreased Na by 21.28% and 20.29%, respectively, under 50 and 100 mM NaCl conditions (Figure 2B).

In this study, salt stress decreased K concentration, while increasing Na concentration in leaves of bitter melon. Potassium uptake is linked to sodium in roots and the excess Na from salt stress decreases K uptake [61]. Potassium has cellular functions in tolerance to stress and is important for regulating transpiration and water uptake [62], carbon dioxide (CO_2_) supply for photosynthesis by stomatal opening, cell expansion, and osmoregulation [63] under salt stress. Here we showed that Cs–Se NPs spray significantly reduced Na and increased K concentration in leaves of bitter melon. In agreement with our results, Gong et al. [64] demonstrated that selenium formed a complex with Na at the root level that prevented Na from being absorbed via a decrease in apoplastic transport across the root and its translocation to the leaves. Se improved activity of plasma membrane H-ATPase, thus significantly reducing Na and increasing K uptake under salt stress conditions [65]. Se application decreased K concentration in roots and enhanced K accumulation of in leaves [9], increased the K^+^/Na^+^ ratio and reduced the accumulation of Na^+^ ions in dill [66] and garlic [53], compared to non-treated plants under salinity stress. Jiang et al. [67] reported that overexpression of the NHX_1_ gene in poplar plants resulted in sequestration of Na^+^ ions in the vacuoles and resistance to salt stress. Entrainment of Na^+^ ions in root vacuoles maintained osmotic balance and facilitated movement of water to the upper parts of the plant. Subramanyam et al. [68] reported that Na_2_SeO_4_ application significantly increased OsNHX_1_ transcript levels in rice under salinity stress condition.

### 3.5. Effects of Cs–Se NPs on Proline and Soluble Sugars in Leaves under Salinity Stress

Salinity significantly increased total soluble carbohydrates and free proline by 52.96% and 38.61%, respectively, compared to non-saline controls. However, application of 20 mg L^−1^ Cs-Se NPs resulted in a larger increase in total soluble carbohydrates by 34.75%, 34.55%, and 28.04% and free proline by 12.30%, 10.08%, and 8.86%, with no salt, 50 and 100 mM NaCl, respectively (Figure 2C,D). Proline is a biocompatible solute that accumulates in plants under stress condition. Szabados and Savoure [69] reported that proline acted as a molecular chaperone to maintain protein integrity. Nawaz et al. [70] showed proline linkage with metal ions useful for defending against damage in stressed plants. One of the key mechanisms for plant tolerance to salinity is accumulation of osmolytes such as free proline and soluble sugars [71]. Ullrich [72] reported that salt stress altered assimilation, accumulation, and metabolism of nitrogen, which plays an essential role in the biosynthesis of proline. In our experiments, salinity caused an accumulation of free proline and total soluble carbohydrates, while application of Cs–Se NPs significantly increased the level of total soluble carbohydrate and proline in bitter melon plants under salt stress. The Se NPs also increased salinity tolerance by stabilizing membranes [73]. Total soluble carbohydrates act as an energy source during salt stress to overcome the perturbation of cellular metabolism and thus maintain growth. Our results are in line with the findings of Zahedi et al. [51] who reported that 10 and 20 mg L^−1^ Se NP foliar spray significantly increased proline and total soluble carbohydrate on strawberry plants under non-saline and salinity stress condition.

### 3.6. Effects of Cs–Se NPs on Leaf MDA and H_2_O_2_ Content and EL under Salinity Stress

**Figure 3:**
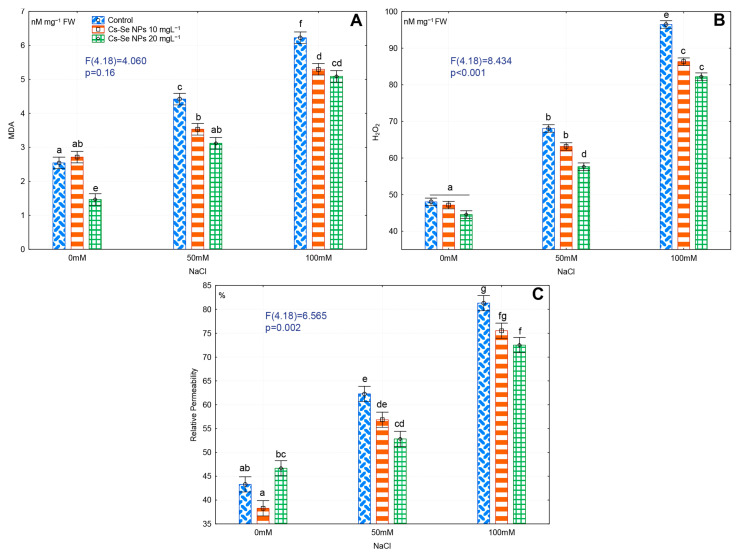
Effect of increasing concentration of Cs–Se NPs on MDA (**A**), H_2_O_2_ (**B**), and relative permeability (%) (**C**) of bitter melon (*Momordica charantia*) leaves under salinity stress. The SDs denoted by lower-case letters in the same treatment indicate significant differences at *p* < 0.05 by Tukey’s post hoc test.

Salinity caused accumulation of MDA, promoted relative electrolyte leakage and H_2_O_2_ levels. The increased MDA, relative electrolyte leakage, and H_2_O_2_ were closely related to the harmful effects of salt stress. Severe salinity significantly increased MDA, relative electrolyte leakage, and H_2_O_2_ by 59.16%, 46.74%, and 50.22%, respectively, compared to non-saline condition, but application of Cs–Se NPs significantly reduced MDA, relative electrolyte leakage and H_2_O_2_ content under salinity conditions. Foliar application of 20 mg L^−1^ Cs–Se NPs significantly reduced the MDA (by 29.47% and 18.32), H_2_O_2_ (by 15.35% and 14.77%), and relative electrolyte leakage (by 15.15% and 10.80%), respectively, under 50 and 100 mM NaCl (Figure 3A–C).

Salinity causes oxidative stress, damages cellular components and can result in cell death by accumulation of high levels of ROS [1]. In our experiments, salt stress increased H_2_O_2_ and MDA content indicating severe oxidative stress. However, application of Cs–Se NPs significantly reduced the salt-induced H_2_O_2_ and MDA increases. Se activates the antioxidant defense system in plants, which lowers cellular H_2_O_2_ and MDA concentrations [74,75]. Feng et al. [74] reported that Na_2_SeO_4_ protected membranes by activating antioxidant enzymes that reduced lipid peroxidation. In agreement with this study, Hawrylak-Nowak [11] in cucumber and Shekari et al. [66] in dill plantlets reported that Na_2_SeO_4_ pretreatment reduced H_2_O_2_ formation under salinity stress. Moreover, Zahedi et al. [51] reported that 10 and 20 mg L^−1^ Se NP foliar spray significantly decreased MDA and H_2_O_2_ on strawberry plants under non-saline and salinity stress condition.

### 3.7. Effects of Cs–Se NPs on Antioxidant Enzyme Activity in Leaves under Salinity Stress

**Figure 4:**
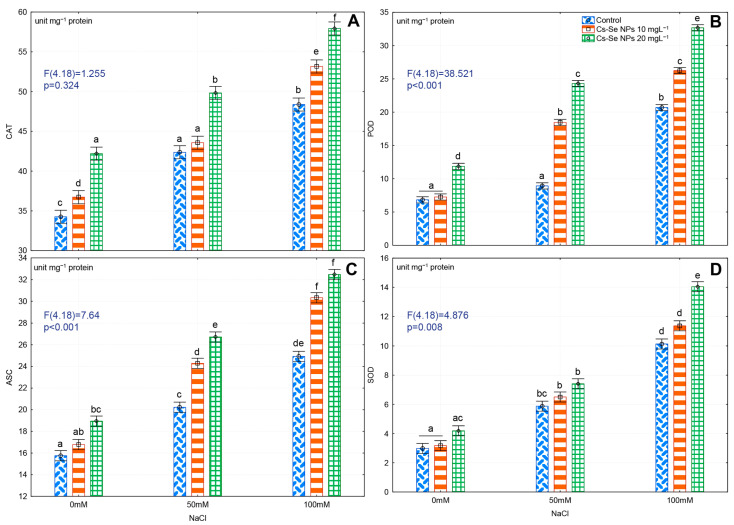
Effect of increasing concentration of Cs–Se NPs on CAT (**A**), POD (**B**), APX (**C**), and SOD (**D**) enzyme activity in bitter melon (*Momordica charantia*) leaves under salinity stress. The SDs denoted by lower-case letters in the same treatment indicate significant differences at *p* < 0.05 by Tukey’s post hoc test.

Plants use antioxidant systems to eliminate ROS. In this study, high salt exposure significantly increased POD, SOD, APX, and CAT activity by 29.19%, 67.03%, 36.79%, and 70.58%, respectively, compared to control plants under normal conditions. As well as, exogenous 20 mg L^−1^ Cs–Se NPs significantly improved CAT, POD, and APX activity by 18.83%, 42.49%, and 16.84% in controls, and CAT by 14.97% and 16.50%, POD by 63.33% and 36.66%, and APX activity by 24.29% and 23.25%, respectively, after exposure to 50 and 100 mM NaCl (Figure 4A–C). Moreover, exogenous 20 mg L^−1^ Cs–Se NPs significantly improved SOD activity by 27.84% with 100 mM NaCl (Figure 4D).

Iqbal et al. [5] reported that application of Na_2_SeO_4_ increased CAT, APX, and SOD activity in various environmental stress conditions. In this study, when bitter melon plants were given a foliar spray with Cs–Se NPs, the CAT, POD, APX, and SOD activities were increased. Shekari et al. [66] stated that Na_2_SeO_4_ application improved the CAT, APX, and SOD activity of *Anethum graveolens* under salt stress. Se increased CAT, SOD, and APX activities by upregulating expression of antioxidant defense genes in maize plants [48]. Moulick et al. [76] showed that Se application improved the translocation of zinc, manganese, and iron into the shoots in rice. Nouet et al. [77] reported that zinc, iron, and manganese were important parts of antioxidant enzymes and enhanced CAT, SOD, and POD activities. Our results show that Cs–Se NPs protected plants from oxidative stress by increasing antioxidant enzyme activity. In line with our results, previous findings show that application of Se NPs increased activity of antioxidant enzymes [16,51].

### 3.8. Effects of Cs-Se NPs on Non-Enzymatic Antioxidant Compounds in Fruit under Salinity Stress

**Figure 5:**
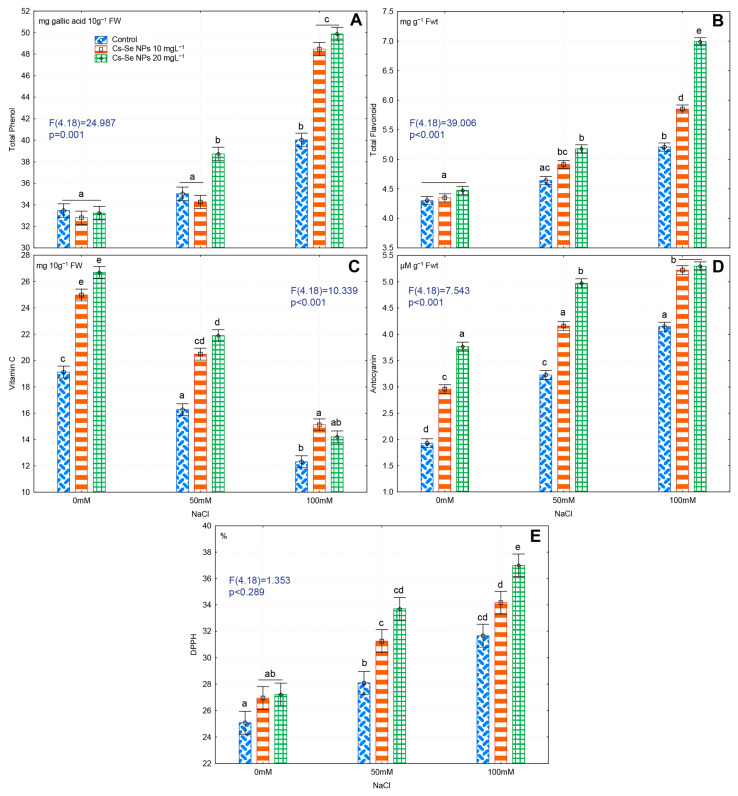
Effect of increasing concentration of Cs–Se NPs on total phenol (**A**), total flavonoids (**B**), vitamin C (**C**), anthocyanin (**D**), and antioxidant activity (DPPH) (**E**) on bitter melon (*Momordica charantia*) fruit under salinity stress. The SDs denoted by lower-case letters in the same treatment indicate significant differences at *p* < 0.05 by Tukey’s post hoc test.

High salinity significantly increased levels of total phenols and flavonoids under salt stress conditions, but foliar application of 20 mg L^−1^ Cs–Se NPs significantly increased the total phenol and flavonoid content even further. At salinity levels of 50 and 100 mM NaCl, 20 mg L^−1^ Cs–Se NPs significantly increased phenols by 9.55% and 19.71% and flavonoids by 10.25% and 25.60%, respectively (Figure 5A,B).

Growth under saline conditions significantly decreased vitamin C content in bitter melon fruit. As observed in this study, salinity decreases photosynthesis in plant. However, biosynthesis of vitamin C required carbohydrate (sugar) production in photosynthesis. Therefore, salinity reduces vitamin C. However, application of Cs–Se NPs increased vitamin C under both non-saline and saline exposure. With exposure to no salt or 50 mM NaCl, foliar treatment with 20 mg L^−1^ Cs–Se NPs significantly increased vitamin C levels by 28.30% and 25.71%, respectively. At a salinity of 100 mM NaCl, 10 mg L^−1^ Cs–Se NPs increased vitamin C levels by 18.53% (Figure 5C). Salt stress and Cs–Se NPs also significantly increased anthocyanin concentration in leaves. In zero saline and plus saline conditions (0, 50, and 100 mM NaCl), foliar treatment with 20 mg L^−1^ Cs–Se NPs significantly increased anthocyanin production by 48.93%, 35.21%, and 21.73%, respectively (Figure 5D). Salinity alone can significantly increase total antioxidant activity in bitter melon fruits; however, foliar application of 20 mg L^−1^ Cs–Se NPs increased total antioxidant activity in the fruits compared to non-treated plants grown in saline soil. At salinity levels of 50 and 100 mM NaCl, treatment with 20 mg L^−1^ Cs–Se NPs significantly increased total antioxidant level by 16.62% and 14.43%, respectively (Figure 5E).

Different types of stress (biotic or abiotic) cause production and accumulation of reactive oxygen species (ROS), which are powerful oxidizing agents that are damaging to cells and can result in interruption of the electron transport chain [1]. Plants use enzymatic and non-enzymatic compounds to remove ROS and protect cells from oxidant damage [78]. Non-enzymatic defenses include compounds, such as phenolic compounds and flavonoids, ascorbate (vitamin C), glutathione, carotenoids, and a-tocopherols (vitamin E) [79]. In accord with our results, other studies have shown that the application of NPs enhanced the level of antioxidants in plants [80]. Multi-walled carbon nanotubes increased total phenolic compounds and essential oil production in basil plants (*Ocimum basilicum*) under salinity stress [81]. ZnO and CuO nanoparticles enhanced the concentration of glycyrrhizin, anthocyanins, total phenolic compounds, and flavonoids in *Glycyrrhiza glabra* [82]. Selenium is a cofactor of antioxidant enzymes, such as glutathione peroxidase, and stimulates production of antioxidants in plants [83]. This property of selenium can be improved when it is applied in the form of NPs with low toxicity [84] and biocompatible physicochemical characteristics NPs [85]. In agreement with our finding Morales-Espinoza et al. [16] reported that application of Se NPs (20 mg L^−1^) increased non-Enzymatic antioxidants compounds such as vitamin C, flavonoids, phenols, lycopene, and β-carotene more than alone salinity in tomato fruit.

### 3.9. Effects of Cs-Se NPs on Essential Oil Yield and Content in Fruit from Plants under Salt Stress

Salinity stress significantly increased essential oil content and the higher the salinity the more essential oil was produced. Foliar treatment with Cs–Se NPs under both non-saline and saline conditions also significantly increased essential oil content. In plants grown on soil with no added salt, the application of 20 mg L^−1^ Cs–Se NPs significantly increased essential oil content by 54.38%. With 50 and 100 mM NaCl, 10 mg L^−1^ Cs–Se NPs significantly increased essential oil production by 17.74% and 15.44%, respectively (Table 3).

Components identified in the oil from bitter melon fruit included: 1- charantin, 2- momordin, 3-momordicoside, 4-momordicin, 5-karaviloside, 6-cucuritan, 7-oleic acid, 8-stigmasterol, 9-cis-9-hexadecenal, 10-cucurbitacin, 11-pentadecyne, 12-gentisic acid. Charantin, momordicoside, karaviloside, gentisic acid, stigmasterol, and oleic acid are important medical compounds. Momordicoside, karaviloside, gentisic acid, oleic acid, and stigmasterol are anticancer compounds and charantin is an anti-diabetic compound. Salinities of 50 and 100 mM had different effects on essential oil composition. In 50 mM NaCl, the concentration of momordin, momordicoside, karaviloside, cucuritan, and oleic acid were increased and in 100 mM NaCl, the concentration of stigmasterol and pentadecyne increased. Salinities of 50 and 100 mM decreased charantin, momordicin, cis-9-hexadecenal, cucurbitacin, and gentisic acid relative to non-saline controls. Foliar treatment with 10 and 20 mg L^−1^ Cs–Se NPs significantly increased charantin, momordicin, oleic acid, and gentisic acid under non-saline conditions, while foliar treatment with 10 mg L^−1^ Cs–Se NPs significantly increased cucurbitacin and stigmasterol and 20 mg L^−1^ Cs–Se NPs significantly increased cis-9-hexadecenal. Treatment with 10 and 20 mg L^−1^ Cs–Se NPs at 50 and 100 mM NaCl significantly increased momordin, momordicin, oleic acid, and stigmasterol. Treatment with 20 mg L^−1^ Cs–Se NPs significantly increased charantin, momordicoside, cucuritan, and gentisic acid at 50 and 100 mM NaCl, karaviloside and cucuritan at 50 mM NaCl, and cis-9-hexadecenal at 100 mM NaCl compared to controls. Treatment with 10 mg L^−1^ Cs–Se NPs significantly increased cucurbitacin concentration at 50 and 100 mM NaCl, charantin, cis-9-hexadecenal and gentisic acid at 50 mM NaCl and momordicoside at 100 mM NaCl compared to controls. Treatment with 20 mg L^−1^ Cs–Se NPs resulted in the highest charantin, oleic acid, cis-9-hexadecenal and gentisic acid content with increases of 50.87%, 47.08%, 22.32%, and 32.01%, respectively, compared to non-treated plants under non-saline conditions. Application of Cs–Se NPs at 10 mg L^−1^ showed highest levels of momordicin (40.99%) and cucurbitacin (38.26%), respectively, compared to non-treated plants under non-saline conditions. Treatment with 20 mg L^−1^ Cs–Se NPs at 50 mM salinity resulted in the highest levels of momordin, momordicoside, karaviloside, and cucuritan with increases of 39.22%, 16.46%, 54.79%, and 24.48, respectively, compared to non-treated plants at 50 mM NaCl salt stress. Foliar treatment with 20 mg L^−1^ Cs–Se NPs at 100 mM NaCl salinity resulted in the highest stigmasterol content with a 20.20% increase compared to non-treated controls (Table 3).

Elevated levels of ROS and cytoplasmic Ca^2+^ and upregulation of mitogen-activated protein kinase (MAPK) are initial responses of plants to NPs. The silver NPs are recognized by plasma membrane-bound receptors and lead to ROS production and a Ca^2+^ burst in *Arabidopsis thaliana* [86]. Application of AgNP leads to upregulated Ca^2+^ levels and associated signaling pathway proteins in *Oryza sativa* roots [87]. These findings show that AgNPs impede cell metabolism by binding to Ca^2+^ receptors, Ca^2+^ channels, and Ca^2+^/Na^+^ ATPases. As sensed by calcium binding proteins (CaBPs) or other NP-specific proteins, NPs may mimic Ca^2+^ or signaling molecules in the cytosol [88]. MAPK phosphorylation and activation of downstream transcription factors results in the transcriptional reprogramming of secondary metabolism in plants [89]. Treatment with TiO_2_ NPs significantly increased essential oil content of *D. moldavica* under salt stress condition [14]. C_60_(OH)_20_ nanoparticles increased the concentration of the antidiabetic insulin and charantin compounds and the anticancer phytochemicals, lycopene and cucurbitacin-B in *Momordica charantia* [90].

**Figure 6:**
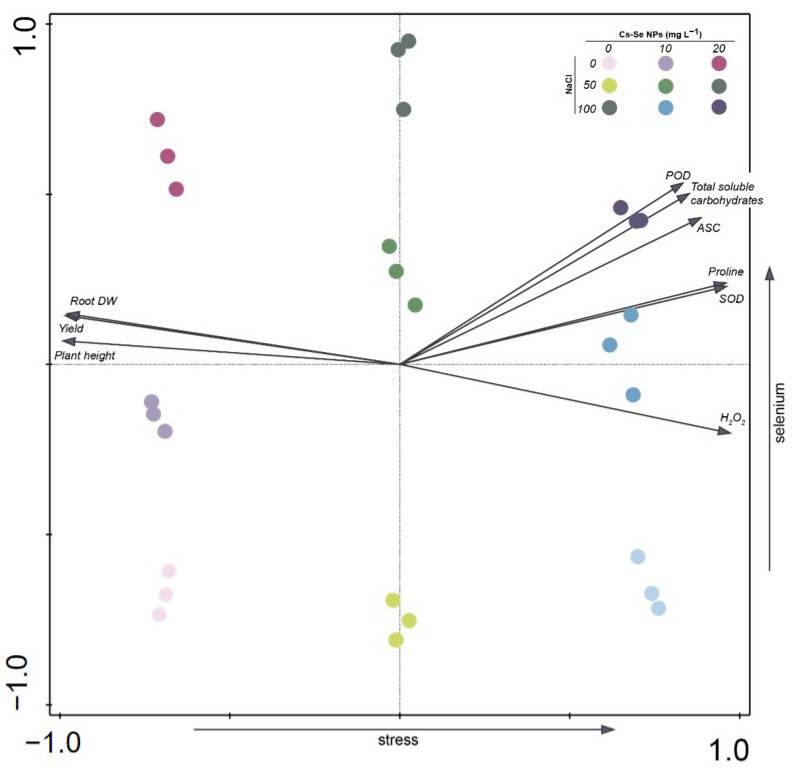
Ordination diagram—PCA (principal component analysis). The bi-plot displays treatments with Cs-Se NPs (0, 10, and 20 mg L^−1^) and salinity stress (0, 50, and 100 mM NaCl). The first and second axes together explain 90.01% of variance. The selenium amount is shown by the color intensity of the circle. The basic color (purple, green, blue) is determined by the salinity stress (table in graph). The arrows show reaction of bitter melon (physiological parameters) under stress.

Individual PCA plots were constructed for bitter melon plants subjected to 0, 50, and 100 mM NaCl salt stress and treated with 0, 10, and 20 mg L^−1^ Cs–Se NPs. The analysis displayed the ten best-fitting variables (plant height, root dry weight, yield, total soluble carbohydrates, proline, hydrogen peroxide (H_2_O_2_), peroxidase (POD), superoxide dismutase (SOD), and ascorbate peroxidase (APX). PCA bi-plots of treatment variable associations showed lines originating from the center indicating positive or negative correlations for different variables. Plant height, root dry weight, and yield had negative correlations and total soluble carbohydrates, proline, H_2_O_2_, POD, SOD, and APX had positive correlations. When bitter melon plants were grown under salinity stress, plant height, root dry weight and yield decreased and total soluble carbohydrates, proline, H_2_O_2_, POD, SOD, and APX increased, while, Cs-Se NPs application increased plant height, root dry weight and yield. Cs-Se NPs application to salt-stressed plants increased total soluble carbohydrates, proline, POD, APX, and SOD more than salinity stress alone. In addition, Cs–Se NPs application under salinity stress conditions decreased H_2_O_2_. Cs–Se NPs improved growth parameters, and increased yield by mobilizing osmotic regulators and antioxidant enzymes, and decreasing oxidative stress (Figure 6). Similar to our results, Jiang et al. [48] reported that Se enhanced plant resistance to salinity stress by increasing synthesis of osmotic regulators, promoting antioxidant activity, and decreasing ROS.

## 4. Conclusions

Nanotechnology is a relatively recent and novel field of study that has great potential for mitigation of plant stress, both biotic and abiotic. Our results showed that application of Cs–Se NPs by foliar spray increased growth and yield of bitter melon plants by increasing expression of photosynthetic pigments in the leaves and raising overall photosynthetic capacity. Application of Cs–Se NPs also enhanced antioxidant enzyme activity and reduced H_2_O_2_ content in leaves, thus decreasing oxidative damage under stress conditions. Cs-Se NPs increased expression of intrinsic antioxidant compounds, such as phenols and flavonoids, ascorbate and anthocyanin, and essential oil content in bitter melon fruits. This result shows a potential treatment for producing bitter melon under salt stress, since the plants will produce fruits of better quality that may be useful as dietary supplements or sources of new drugs.

## Figures and Tables

**Table 1:** Effect of foliar application of Cs–Se NPs (0, 10, and 20 mg L^−1^) on some morphological parameters of bitter melon (*Momordica charantia*) under salt stress (0, 50 and 100 mM NaCl). Data are the mean of 3 replications (n = 3) ± SD, statistically significant difference is given by letters according to the *TMRT* (*p* < 0.05).

NaCl (mM)	Treatments	Plant Height (cm)	Shoot FW (g)	Shoot DW (g)	Root FW (g)	Root DW (g)	RWC (%)	No. Fruits/Plant	Fruit Weight (kg)	Yield (kg)
0	Control	3.97 ± 0.09 a	240.33 ± 16.31 a	65.95 ± 4.17 a	155.69 ± 1.00 a	27.89 ± 0.17 b	91.17 ± 2.98 b	21 ± 0.81 b	0.071 ± 0.003 a	1.501 ± 0.016 b
	Cs–Se NPs 10 mg L^−1^	3.99 ± 0.04 a	242.73 ± 6.03 a	67.25 ± 3.24 a	156.54 ± 1.66 a	28.06 ± 0.28 ab	92.56 ± 1.71 ab	23 ± 0.81 a	0.06 ± 0.002 b	1.470 ± 0.020 b
	Cs–Se NPs 20 mg L^−1^	4.07 ± 0.06 a	246.84 ± 15.02 a	69.60 ± 2.18 a	158.32 ± 1.41 a	28.81 ± 0.51 a	95.98 ± 1.47 a	22.66 ± 0.47 a	0.075 ± 0.002 a	1.702 ± 0.015 a
50	Control	2.51 ± 0.11 c	143.77 ± 9.66 bc	48.48 ± 2.71 bc	107.26 ± 1.78 c	19.25 ± 0.47 d	72.26 ± 1.61 de	14.33 ± 0.47 d	0.052 ± 0.001 c	0.747 ± 0.010 e
	Cs–Se NPs 10 mg L^−1^	2.67 ± 0.04 b	166.11 ± 6.70 b	49.27 ± 0.73 bc	123.48 ± 3.76 b	22.16 ± 0.48 c	76.56 ± 0.93 d	15.66 ± 0.94 cd	0.053 ± 0.006 c	0.827 ± 0.053 d
	Cs–Se NPs 20 mg L^−1^	2.70 ± 0.05 b	173.17 ± 11.26 b	52.21 ± 0.87 b	124.14 ± 1.15 b	22.28 ± 0.34 c	83.50 ± 3.24 c	16.33 ± 0.47 c	0.061 ± 0.001 b	0.996 ± 0.004 c
100	Control	1.22 ± 0.05 e	89.37 ± 5.43 e	38.72 ± 0.65 e	69.01 ± 2.51 e	12.39 ± 0.42 f	53.81 ± 2.17 f	9 ± 0.81 f	0.035 ± 0.002 e	0.316 ± 0.026 g
	Cs–Se NPs 10 mg L^−1^	1.34 ± 0.04 de	108.23 ± 4.52 de	40.71 ± 0.83 de	73.92 ± 4.95 de	13.27 ± 0.77 e	70.70 ± 2.69 e	11 ± 1.41 e	0.032 ± 0.003 e	0.352 ± 0.014 g
	Cs–Se NPs 20 mg L^−1^	1.41 ± 0.05 d	117.94 ± 2.08 d	44.69 ± 1.09 cd	76.93 ± 0.71 d	13.83 ± 0.11 e	74.26 ± 0.84 de	10 ± 1.41 ef	0.043 ± 0.006 d	0.422 ± 0.010 f

**Table 2:** Effect of foliar application of Cs–Se NPs (0, 10, and 20 mg L^−1^) on photosynthetic pigments (Chl a, b, total Chl, and carotenoids) and net photosynthesis rate (Pn) of bitter melon (*Momordica charantia*) under salt stress (0, 50, and 100 mM NaCl). Data are the mean of 3 replications (n = 3) ± SD, statistically significant difference is given by letters according to the *TMRT* (*p* < 0.05).

NaCl (mM)	Treatments	Chl a (mg g^−1^ FW)	Chl b (mg g^−1^ FW)	Total Chl (mg g^−1^ FW)	Carotenoids (mg g^−1^ FW)	Pn (µmol m^−2 ^s^−1^)
0	Control	11.40 ± 0.45 b	4.39 ± 0.11 b	15.80 ± 0.45 b	0.780 ± 0.01 b	9.60 ± 0.23 bc
	Cs–Se NPs 10 mg L^−1^	11.98 ± 0.46 ab	4.44 ± 0.08 b	16.42 ± 0.43 ab	0.820 ± 0.03 ab	9.93 ± 0.14 b
	Cs–Se NPs 20 mg L^−1^	12.65 ± 0.70 a	4.79 ± 0.14 a	17.44 ± 0.57 a	0.835 ± 0.03 a	10.67 ± 0.25 a
50	Control	8.19 ± 0.82 de	3.03 ± 0.05 d	11.22 ± 0.87 e	0.565 ± 0.00 d	8.50 ± 0.16 ef
	Cs–Se NPs 10 mg L^−1^	8.93 ± 0.65 d	3.22 ± 0.15 d	12.40 ± 0.58 d	0.591 ± 0.01 d	8.85 ± 0.08 de
	Cs–Se NPs 20 mg L^−1^	10.27 ± 0.59 c	3.47 ± 0.09 c	13.49 ± 0.44 c	0.669 ± 0.02 c	9.22 ± 0.12 cd
100	Control	5.27 ± 1.19 g	1.89 ± 0.06 g	7.17 ± 1.17 g	0.430 ± 0.09 f	7.26 ± 0.14 h
	Cs–Se NPs 10 mg L^−1^	6.59 ± 0.66 f	2.18 ± 0.12 f	8.78 ± 0.72 f	0.478 ± 0.00 e	7.94 ± 0.20 g
	Cs–Se NPs 20 mg L^−1^	7.68 ± 0.54 e	2.58 ± 0.21 e	10.26 ± 0.70 e	0.517 ± 0.00 e	8.17 ± 0.14 g

**Table 3:** Effect of foliar application of Cs–Se NPs (0, 10, and 20 mg L^−1^) on essential oil content and composition of bitter melon (*Momordica charantia*) fruit from plants grown with 0, 50, and 100 mM NaCl. Data are the mean of 3 replications (n = 3) ± SD, statistically significant difference is given by letters according to the *TMRT* (*p* < 0.05).

NaCl (mM)	Treatments	Oil content (%)	Charantin	Momordin	Momordicoside	Momordicin	Karaviloside	Cucuritan	Oleic acid	Stigmasterol	Cis-9-hexadecenal	Cucurbitacin	pentadecyne	Gentisic acid
0	Control	0.380 ± 0.02 f	1.40 ± 0.07 c	0.643 ± 0.19 e	0.341 ± 0.00 d	0.498 ± 0.01 c	0.787 ± 0.08 bc	0.495 ± 0.01 bcd	0.700 ± 0.07 cd	1.07 ± 0.08 f	2.47 ± 0.06 c	1.42 ± 0.32 bcd	9.76 ± 1.47 a	6.35 ± 0.34 c
	Cs–Se NPs 10 mg L^−1^	0.480 ± 0.02 f	1.84 ± 0.10 b	0.623 ± 0.14 e	0.348 ± 0.01 d	0.844 ± 0.00 a	0.667 ± 0.01 c	0.484 ± 0.00 bcde	1.316 ± 0.13 a	1.41 ± 0.09 de	2.36 ± 0.06 c	2.30 ± 0.57 a	8.51 ± 1.37 ab	7.43 ± 0.20 b
	Cs–Se NPs 20 mg L^−1^	0.833 ± 0.01 e	2.85 ± 0.10 a	0.566 ± 0.19 e	0.348 ± 0.01 d	0.816 ± 0.00 a	0.682 ± 0.01 c	0.532 ± 0.00 bc	1.323 ± 0.10 a	1.20 ± 0.08 ef	3.18 ± 0.07 a	1.96 ± 0.10 ab	5.66 ± 1.41 b	9.34 ± 0.24 a
50	Control	1.02 ± 0.04 d	1.12 ± 0.08 de	1.10 ± 0.03 cd	0.487 ± 0.00 b	0.314 ± 0.01 d	0.877 ± 0.07 b	0.549 ± 0.08 b	0.740 ± 0.09 bcd	1.21 ± 0.12 ef	1.91 ± 0.09 e	0.91 ± 0.17 cd	10.72 ± 1.50 a	5.71 ± 0.26 de
	Cs–Se NPs 10 mg L^−1^	1.24 ± 0.07 abc	1.55 ± 0.09 c	1.51 ± 0.08 b	0.501 ± 0.01 b	0.786 ± 0.00 a	0.786 ± 0.01 bc	0.474 ± 0.00 cde	0.916 ± 0.05 b	1.65 ± 0.21 d	2.27 ± 0.11 cd	1.56 ± 0.23 b	9.84 ± 1.32 a	6.45 ± 0.30 c
	Cs–Se NPs 20 mg L^−1^	1.17 ± 0.05 bc	1.92 ± 0.06 b	1.81 ± 0.02 a	0.583 ± 0.02 a	0.695 ± 0.00 b	1.94 ± 0.05 a	0.727 ± 0.00 a	0.906 ± 0.08 b	1.97 ± 0.08 c	2.06 ± 0.07 de	1.66 ± 0.14 b	8.21 ± 1.50 ab	7.24 ± 0.23 b
100	Control	1.15 ± 0.04 c	0.966 ± 0.06 ef	0.856 ± 0.09 de	0.211 ± 0.02 e	0.163 ± 0.01 e	0.770 ± 0.05 bc	0.392 ± 0.00 f	0.663 ± 0.08 d	2.29 ± 0.22 b	2.39 ± 0.11 c	0.862 ± 0.11 d	11.74 ± 1.51 a	5.21 ± 0.26 e
	Cs–Se NPs 10 mg L^−1^	1.36 ± 0.04 a	0.916 ± 0.04 f	1.17 ± 0.04 c	0.430 ± 0.01 c	0.298 ± 0.01 d	0.773 ± 0.06 bc	0.417 ± 0.01 ef	0.870 ± 0.07 bc	2.68 ± 0.05 a	1.89 ± 0.04 e	1.50 ± 0.25 bc	11.66 ± 1.33 a	5.17 ± 0.31 e
	Cs–Se NPs 20 mg L^−1^	1.29 ± 0.08 ab	1.20 ± 0.06 d	1.35 ± 0.12 bc	0.518 ± 0.01 b	0.347 ± 0.11 d	0.846 ± 0.08 b	0.449 ± 0.03 def	0.860 ± 0.04bc	2.87 ± 0.08 a	2.76 ± 0.19 b	0.868 ± 0.06 d	11.04 ± 1.53 a	5.83 ± 0.21 cd

## Data Availability

The data that support the findings of this study are available from the corresponding author upon reasonable request.

## Abbreviations

Cs, chitosan; Se: Selenium; Cs–Se NPs, chitosan-selenium nanoparticles; FW, fresh weight; DW, dry weight; RWC, Relative water content; EL, electrolyte leakage; H2O2, hydrogen peroxide; POD, peroxidase; CAT, catalase; SOD, superoxide dismutase; APX, ascorbate peroxidase; Chl, chlorophyll; MDA, malondialdehyde.
